# Innate and Cultural Spatial Time: A Developmental Perspective

**DOI:** 10.3389/fnhum.2017.00215

**Published:** 2017-05-03

**Authors:** Barbara Magnani, Alessandro Musetti

**Affiliations:** ^1^Private PractitionerReggio Emilia, Italy; ^2^Department of Humanities, Social Sciences and Cultural Industries, University of ParmaParma, Italy

**Keywords:** spatial map of time, magnitude system, mental time line, metaphorical concepts, mental number line

## Abstract

We reviewed literature to understand when a spatial map for time is available in the brain. We carefully defined the concepts of metrical map of time and of conceptual representation of time as the mental time line (MTL) in order to formulate our position. It is that both metrical map and conceptual representation of time are spatial in nature. The former should be innate, related to motor/implicit timing, it should represent all magnitudes with an analogic and bi-dimensional structure. The latter MTL should be learned, available at about 8–10 years-old and related to cognitive/explicit time. It should have uni-dimensional, linear and directional structure (left-to-right in Western culture). We bear the centrality of the development of number cognition, of time semantic concepts and of reading/writing habits for the development of ordinality and linearity of the MTL.

## Introduction

**Figure 1:**
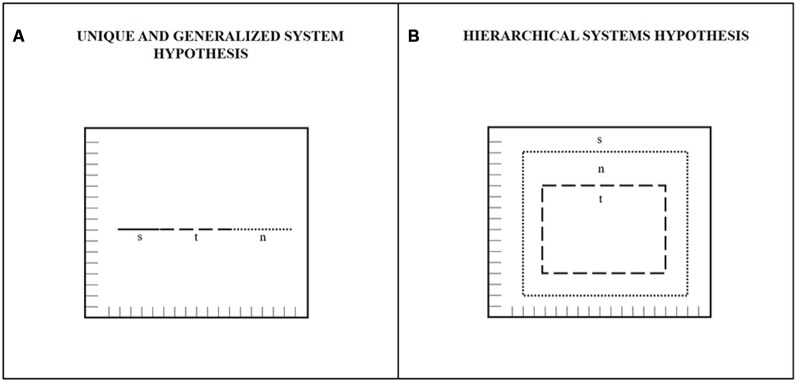
**Graphical representations of: (A)** Unique and generalized system of magnitude representations. This system is represented as a unique metrical space completely shared by all quantities (s = space; t = time; n = numbers) where any manipulation of one dimension should interfere with the representation of other dimensions. In this system interference among dimensions should be symmetrical. **(B)** Hierarchical systems of representations. This system is represented as a spatially and hierarchically overlapped maps system where manipulations of s should interfere with the representations of n and t, the manipulation of n should interfere with t but not with s and the manipulation of t should not interfere with n and s.

Numerous studies agree that the brain maps time dimension by a spatial code (Walsh, 2003; Bueti and Walsh, 2009; Oliveri et al., 2009). They are based on A Theory Of Magnitude (ATOM—Walsh, 2003; Bueti and Walsh, 2009). ATOM assumes that space, time and other quantities (i.e., numbers) rely upon an innate and generalized magnitude system that computes representations such as “less than–more than”, “slower–faster”, “nearer–farther”, “smaller–bigger” useful for action. The paradigm used to support that the system for representing magnitude is unique and generalized among magnitudes is that of symmetrical or asymmetrical cross-dimensional influences (Dormal et al., 2006, 2008). If the magnitude system is unique and generalized, we should appreciate symmetrical influence for example between spatial and temporal stimulation or between numerical and spatial stimulation or between temporal and numerical stimulation and so on. In other words, if the system is one and only one, the same system employed for the processing of one dimension cannot be employed for the processing of another dimension at the same time. If this is the case, a spatial task should be influenced by a temporal stimulation with the same degree to which a temporal task should be influenced by a spatial stimulation. Equally, a numerical task should be influenced by a temporal stimulation with the same degree to which a temporal task should be influenced by a numerical stimulation and so on with all combinations of magnitudes. On the contrary, asymmetrical influences among magnitudes support the existence of multiple systems instead of just a one that are partially dependent among each other. If a spatial stimulation interferes with a temporal task but the reverse does not happen, we can assume that temporal system does depend on spatial system but the spatial system does not depend on the temporal system. Again, if a numerical stimulation interferes with a temporal task but the reverse does not happen, we can assume that temporal system does depend on numerical system but the numerical system does not depend on the temporal system. Studies supporting a unique system by showing symmetrical influences (Galton, 1880; Dehaene et al., 1993; Droit-Volet, 2003; Dormal et al., 2006, 2008; Torralbo et al., 2006; Vicario et al., 2007, 2008; Ishihara et al., 2008; Umiltà et al., 2009; Vallesi et al., 2011) and studies supporting partially dependent systems by showing asymmetrical influences (Castelli et al., 2006; Casasanto and Boroditsky, 2008; Bottini and Casasanto, 2013; Droit-Volet and Coull, 2015) are equally present in literature. For the former case, for example, Merritt et al. (2010) trained two rhesus macaques to classify lines on their duration or spatial extent by means of a touch screen. One macaque was trained to classify the durations first, while the other macaque was trained to classify the lengths first. After the training on durations or lengths, both macaques underwent the classification task in the reverse condition. Results showed a similar sensitivity to durations and lengths in both macaques and the degree to which durations and lengths influenced the classification of one another was the same. For the latter case, for example, asymmetries have been demonstrated where space always interferes with numbers and time, numbers always interfere with time but rarely with space and time rarely interferes with numbers and space (Cappelletti et al., 2009). In the same work by Merritt et al. (2010), asymmetries between space and time were found in an identical task as described for macaques but in humans. That is, the influence of length on duration classifications was bigger than the influence of duration on length classifications. In contrast, Mendez et al. (2011) found asymmetries indicating the influence of length on duration bigger than the influence of duration on length, not in humans but in a primate. Studies on the interaction between time and numbers have so far reported unidirectional influence with time processing more often affected by number processing (e.g., Droit-Volet, 2003; Dormal et al., 2006, 2008). Studies in favor of partially overlapped systems do not only contrast the existence of an innate and generalized system but also suggest a hierarchical organization of them where the representation of space seems the more salient one, the representation of time seems the less salient one and the representation of numbers seems located in the middle (see Figure 1 for a graphical representation of the unique and the hierarchical systems hypothesis).

The present review inserts inside the debate about ATOM theorization of magnitude system trying to clarify some misunderstanding under this confusion. Our intention is to contribute to doubts about the generalized or the hierarchical organization of spatial maps for quantities. Clarification about the generalized or the hierarchical organization of quantities maps would also insert inside the debate about the innate or the cultural nature of the spatial representations of quantities (Proctor and Cho, 2006; Bonato et al., 2012, 2016 for the debate on the spatial nature of quantities representations).

## Which Spatial Map for Which Time?

### Definitions of Spatial Map

With spatial map, we mean the code used by the brain to deal with time. We refer to “mental time line (MTL)” as the functional product of cognitive operations on time where temporal stimuli are represented with a linear ascending ordinality (Oliveri et al., 2009; Bonato et al., 2012). With linearity, we refer to the displacement of the representation of time on a line (we do not refer to the mathematical function on which temporal judgments distribute—see Wearden and Lejeune, 2008 for explanations on linear or scalar functions of timing). With ascending ordinality, we mean the order (from the smaller to the larger for example) that stimuli representations assume on the line. Support to this ordered organization comes from demonstration that the left hand responds faster to short intervals while the right hand responds faster to long intervals (Conson et al., 2008; Vallesi et al., 2008; Oliveri et al., 2009). The spatial position of temporal stimuli influences their encoding: stimuli presented on the left of space are encoded as shorter than stimuli presented on the right (Vicario et al., 2008). Shifting spatial attention to the left shortens time durations while shifting spatial attention to the right lengthens time durations (Vicario et al., 2008; Frassinetti et al., 2009; Magnani et al., 2011). Time concepts such as days of the week, months, years (Gevers et al., 2003, 2004) or past and future (Torralbo et al., 2006; Santiago et al., 2007) are also ordered in a left-to-right fashion. In an interesting work by Ouellet et al. (2010a) the mere presentation of past and future words was sufficient to orient attention and to prime motor responses with left hand for past words and right hand for future words. Similarly, the same group (Ouellet et al., 2010b) instructed Spanish participants to respond to temporal reference of verbs and adverbs referring to either past or future. Verbs and adverbs were auditorily presented to the left or right ear and participants responded by pressing a left or a right key. Spanish participants responded faster to past words with the left hand and to future words with the right hand. Here the question is whether the spatial code employed to process time is always linear in nature and structured in an ascending order from left to right. Studies on different cultures suggest that the left-to-right MTL is a feature of Western individuals and that Chinese and Indian individuals adopt a circular map for time concepts (Tordjman, 2015). In his works Tordjman refers particularly to the subjective construct of time and to the attitude to focus on the present time as in some cultures or neural disease as autism (Tordjman, 2015), an attitude that tends to synchronize with circadian rhythm, and not on the tendency to order more abstract time concepts. Anyway, even if we consider the tendency to order abstract time concepts, we found cultural differences. Núñez and Sweetser (2006) observed that the Aymara talk and make gests about the future as behind them and the past as ahead of them. Mandarin speakers more likely use vertical metaphors (MT) for time than English speakers do (Scott, 1989; Chun, 1997; Chen, 2007). Fuhrman and Boroditsky (2010) found that English speakers were faster for “earlier” judgments with the left response key than with the right response key, while Hebrew speakers showed exactly the reverse pattern. On a similar vein, the same study described above on the association between the left hand and past words and the right hand and future words in Spanish participants (Ouellet et al., 2010b) demonstrated an opposite pattern for Hebrew participants that show a right-to-left reading habits, with right hand and past words and left hand and future words associations. Another study underlines that Israeli individuals (with a right-to-left reading habits) do not show any association between left hand and short intervals and right hand and long intervals as Italian individuals do (Vallesi et al., 2014). These differences in the displacement and the direction of the MTL in different cultures suggest that cultural factors favor such a directionality in line with the Metaphor Theory (MT—Lakoff and Johnson, 1999). It poses that cross-domain representations use the representation of space to structure metaphorically the domain of numbers and time. There would be not a prototypical generalized system for quantities as theorized by ATOM but metaphorical concepts would be shaped on the representation of space. These theories, apparently in contrast, are proposed to be complementary (Winter et al., 2015) suggesting the existence of a generalized system (ATOM) for simple interactions and more complex systems for more complex MT.

Another way to reason about ATOM and MT differences could be to clearly define the concepts of magnitude map and metaphorical-conceptual representations.

By magnitude map, we mean a representation with a bi-dimensional metric structure. A metric structure has the property of determining binary relations between elements (Montemayor and Balci, 2007). The binary relation imposes that if A is larger than B, B is not larger than A. In order to compute this binary relation, the representation of a metric space with a global distance function between two points is necessary. In simpler words, a magnitude (metric) relation between two elements (for example, A and B) implies that we can determine whether A is larger or smaller than B based on the distance of two points in the metric space. A representation is a magnitude map if we do not need the semantic meaning of A and B to determine the binary relation and if such a relation is context-independent (Montemayor and Balci, 2007). In other words, if we have the representation of the extension of A and B, we do not need to know their quality content (space, time, numbers, luminosity or weight?) to determine which is the larger of the two. On the contrary, a representation is metaphorical-conceptual if, and only if, it requires the existence of constituents with syntactic and semantic properties (compositional rules, Montemayor and Balci, 2007). For example, concepts such as “before” and “after” or “few” and “most” are quantifiers but they need syntactic rules and they depend on the context to acquire their meaning of value. The meaning of “few” cannot be determined by the representation of a binary relation between distances of two points. “Before” could mean “first” or “second” if we are speaking about who arrived before the “third” in a race. “Few” could mean “three” if we are speaking about euros to buy a car or “twenty” if we are speaking about abs we are going to do in our workout. In order to compute compositional rules, a uni-dimensional, linear and ordered representation could be proper to favor the attribution of the meaning.

The definition of magnitude and metaphorical-conceptual representations on the one hand clarifies some confusion, on the other hand raises questions: are ATOM and MT simple epiphenomena of representation definitions, that is, are the two theories simply descriptions of different representations? An alternative question could be: are there two distinct processes for magnitude maps (a common metrical space as for ATOM) and for metaphorical-conceptual representations (as for MT)? Our hypothesis is that ATOM and MT theories describes two processes for two kind of time representations.

### Definitions of Time

Time processing is a complex function (Joireman and Strathman, 2005; for digressions on psychological time, timing or time perception—Meck, 2003; Joireman and Strathman, 2005; Glicksohn and Myslobodsky, 2006; Grondin, 2008). Numerous efforts are made to understand whether time processing depends on a unique and articulated neural system or on multiple systems (Salvioni et al., 2013). For example, Coull and Nobre (2008) defined *implicit* and *explicit* timing as two distinct processes (Zelaznik et al., 2005). Implicit timing would require temporally structured sensorimotor information, without a specific instruction to pay attention to time (i.e., predicting the time a kicked ball will arrive to our hands). Explicit timing would require a deliberate estimation of a duration (Droit-Volet and Rattat, 2006; Magnani et al., 2012, 2013). Another similar distinction is that between *brief* and *long* intervals to be timed (Lewis and Miall, 2003). Brief intervals (undersecond) would be proper of motor control (Arshavsky et al., 1978, 1997; Passingham, 1996; Lewis and Miall, 2002), while long intervals (suprasecond) would be proper of cognitively controlled time (Lewis and Miall, 2003, 2006). These attempts to subdivide temporal processes appear inconsistent for empirical factors. It is difficult to describe what would happen whether a task required responding to the sum of two brief intervals that resulted in a long interval (see Montemayor and Balci, 2007, for detailed description of compositionality rules in cognitive representations). Which process would be employed in this case? Implicit or explicit? Motor or cognitive? Another empirical factor that makes such distinctions apparently inconsistent could be that we can compute cognitive and explicit operations on very brief intervals and motor or implicit operations on very long intervals. Anyway, they can reflect different patterns for the processing of magnitude map and metaphorical representation. Instead of considering these processes as categorial and dissociated with each other, we can consider them as poles of a continuum. We could say that the more the time task is implicit, the more a magnitude representation (innate and common for all magnitudes as for ATOM, Bueti and Walsh, 2009) would be involved and, in turn, motor and primary sensory neural components are required. Similarly, the more the time task requires explicit attention to time or cognitive processes on the time representation, the more metaphorical-conceptual representations would be involved with compositionality rules (syntactic, semantic and context-dependent) and, in turn, cortical structure are required. In practical words, even if to take a kicked ball we compute complex behaviors and movements, we probably do not need to displace the duration of the ball trajectory on a linear and ordered representation, but a metrical map to represent the duration extension, the weight of the ball and the space of the trajectory would be more appropriate. By contrast, if we have to structure a memory in the past or to imaging the future or if we have to take temporal decision for making predictions, a linear and ordered representation would be more appropriate. In line, there is demonstration that the magnitude map of time is controlled by subcortical or sensory structures while the metaphorical-conceptual representation is controlled by neo-cortical structures (Ghose and Maunsell, 2002; Lewis and Miall, 2003).

This clarification helps us to investigate more about *when the spatial map for time develops in the brain*. After theories described so far, we propose that both the metrical map and the metaphorical-conceptual representation of time would be spatial in nature. The metrical map is spatial by definition since we need the representation of a metric space and a binary relation on distances to define it. Moreover, the same metric space is involved independent of the quality of the magnitude (i.e., space, time, numbers, luminosity or weight and so on). However, the metrical map does not have peculiar shape or properties of ordinality or linearity since we need compositionality rules (syntactic, semantic and context-dependent) for such properties. The metaphorical-conceptual representation, even if composed by compositional rules, should assume spatial shape, ordinality and linearity by the context-dependent meaning of constituents (before-after, few-most). The linearity and ordinality of number representation and of reading/writing habits in different cultures would contribute to provide the “spatial context” to structure the linearity and ordinality of the metaphorical-conceptual representation of time. Thus, we can answer our question saying that *both* magnitude metrical map and metaphorical representations are spatial, even with different spatial features. To reformulate, our hypothesis is that a spatial metrical map for time is innate and generalized among magnitudes (Walsh, 2003) and that the spatial meaning of shape, ordinality and linearity of the metaphorical-conceptual time representation is learnt, cultural and grows up along with number cognition and reading/writing habits.

## Development from a Spatial Non-Linear Map of Time to the Mental Time Line

### Innate Map for Quantities

The presence of an innate magnitude system is supported by demonstrations that infants can discriminate quantities such as three vs. two and sometimes four vs. three but not four vs. five (Gallistel and Gelman, 2000). An explanation is that infants (and animals) estimate small quantities by a perceptual process (named “subitizing”) that does not involve counting and cannot represent more than about four objects at a time (Davis and Pérusse, 1988; Trick and Pylyshyn, 1994; Gallistel and Gelman, 2000). Associative learning across magnitudes in 9-month-olds and cross-dimensional transfer occurring symmetrically for all combinations of size, numerosity and duration provides further support for an early and pre-linguistic magnitude system (Lourenco and Longo, 2010). Similar symmetrical influence among quantities has been frequently found in animals (Church and Meck, 1984; Brannon and Roitman, 2003) and in children in kindergarten (Levin, 1977) supporting the existence of an innate system equipped with one spatial-analogic dimension that can compute quantities until cognition is developed. A spatial metrical map for time allows simple operations on time that does not necessarily require compositional rules or a left-to-right orientation. This is the case of the time bisection task (Wearden, 1999) where participants classify a range of durations as more similar to a “short” or a “long” previously learned stimulus (Wearden, 1999). To classify intervals as short or long a metrical map of the interval would be involved. Here, one should ask why a metrical map would be involved and not a conceptual representation of time since cognitive operations are required (learning and memory). The point is that even if memory processing is involved to learn temporal standard durations, the comparison between two durations can be computed in terms of global extension and binary relation on two points distances. Numerous researches confirm that animals like pigeons (Johnson et al., 2010; de Carvalho et al., 2016) and 3-year-old children are able to perform this task (Droit-Volet and Wearden, 2001, 2002). Moreover, Droit-Volet et al. (2004) demonstrated that 5-year-old children are able to perform this task but their time performance is not influenced by a stimulation with arrows indicating left or right direction. On the contrary, the time performance of 8 and 10 year-old children is influenced by arrows stimulation. Again, one should ask why a spatial stimulation does not interfere with the supposed metrical map in young children if the metrical map is spatial in nature. We can notice that the spatial stimulation employed in this work is a symbolic spatial stimulation (arrows indicating left or right direction) that acquire their spatial meaning later in the development. It would be interesting to look for interference between space and time in young children where the spatial stimulation is the extent of a spatial stimulus (i.e., length of a line) instead of a symbolic spatial stimulus. Indeed, authors concluded that the temporal distortions induced by symbolic representations of space (arrows) emerges with the development at around 8–10 years-old. Not surprisingly, 8–10 year-old children reach adult-like levels of sensitivity to time (Droit-Volet and Wearden, 2001). These results are in favor of an innate metrical map of time for simple time operations that is not distorted by symbolic stimulation that evolves in a metaphorical-conceptual representation of time acquired with the cognition development. The metaphorical-conceptual representation would be available at about 8–10 years-old and would be structured on the concepts spatial meaning as demonstrated by the interference of the spatial meaning (indicating directionality i.e., left or right) on the processing of time.

### Cultural Representation of Time

The spatiality of the meaning of conceptual representations of time is evident in metaphorical tasks testing for space-time interactions in 4–10 year-old children where participants result systematically better in judging space than time and space processing always interferes with time processing. Moreover, the language has a role in the precision of the judging of both space and time *per se* (Bottini and Casasanto, 2010; Casasanto et al., 2010). This suggests that the semantic component of language plays a crucial role in shaping an ordered conceptual representation of time. We often talk about time using words with spatial primary meaning, such as short/long, back/forward or up/down (Clark, 1973; Bottini and Casasanto, 2010). The semantic component of language would give ordinality to time concepts by attributing a semantic meaning to time with spatial properties translatable in a visual-like-imagery.

In line with the importance of numerical cognition for the ordinality and linearity of conceptual representations of time, we know that in children numerical information interferes with duration discrimination, but not vice-versa (Droit-Volet et al., 2003; Dormal et al., 2006). This suggests that number processing is more automatic than time processing (Droit-Volet et al., 2003) and probably antecedent in the development. Droit-Volet et al. (2004) submitted 5, 8 and 10 year-old French participants to the classic time bisection task in two conditions: a spatial condition (arrows stimuli) and a numerical condition (digit stimuli). Children aged 5 years showed a lack of interference from space to time and a complete disruption of temporal performance during numerical condition. The effect of association between low digits and short intervals and high digits and long intervals emerged at 8 years-old, while the effects of left arrows on short intervals and right arrows on long intervals emerged between 8 years-old and 10 years-old. To authors, this dissociation might reflect the different steps at which children integrate symbolic (conceptual) representations of number (digits) and of spatial meaning (arrows). Until a symbolic representation of numbers is not acquired as after 5 years-old, digits may be more salient and difficult to inhibit to accurately judge time (Droit-Volet et al., 2004). Studies supporting the centrality of the development of the linear number representation for temporal cognition are numerous (Roitman et al., 2007; Bonato et al., 2012; Droit-Volet and Coull, 2015; Winter et al., 2015), in line with the supposed hierarchy of the conceptual representations development from space to time passing across numbers.

Anyway, the directionality of the mental number line (MNL) does not appear strong enough to structure the directionality of the MTL. If this was the case, Vallesi et al. (2014) should have found the same spatial–temporal association of response codes (STEARC) effect, with left-short and right-long associations, in Italian and Israeli individuals. Despite their right-to-left reading/writing habits, Israeli individuals show a left-to-right MNL. The null STEARC effect found in Israeli individuals vs. the left-to-right STEARC effect found in Italians could reflect an interaction between MNL and reading/writing habits directionality in shaping the MTL directionality. In line, Shaki et al. (2009) found: (i) a strong left-to-right spatial–numerical association of response codes (SNARC) effect in Canadian individuals where both the MNL and the reading/writing habits are left-to-right oriented; (ii) a strong right-to-left SNARC effect in Arabic individuals where both the MNL and the reading/writing habits are right-to-left oriented; and (iii) a null SNARC effect in Israeli individuals where the MNL is left-to-right oriented while reading/writing habits are right-to-left oriented. The interaction between MNL and reading/writing habits for the directionality of time representation is supported by the results of Tversky et al. (1991). They found that English children represented time from left to right, while Arabic children represented time from right to left and, finally, Israeli children did not show any directionality in the representation of time.

## Conclusions

**Figure 2:**
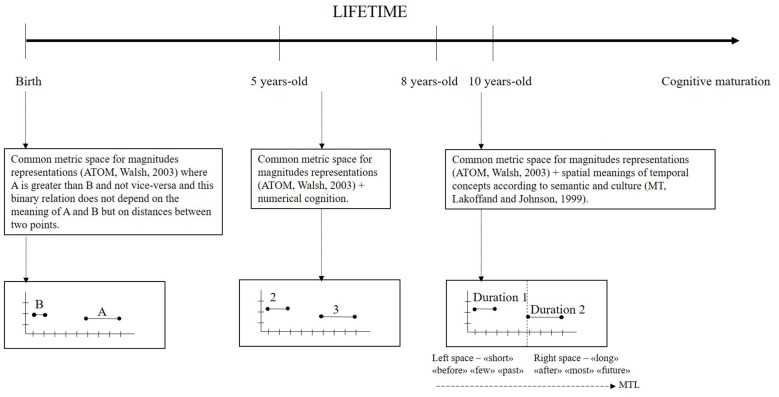
**Graphical representation of the development of the mental time line (MTL; dashed horizontal arrow) in the lifetime as explained in the present review.** Vertical lines on the lifetime arrow sign the principal age steps discussed in the main text (innate, 5, 8 and 10 years-old) and squares indicated by dashed vertical arrows contain explanations and their correspondent graphical explanation.

We selected and revised literature to address the question: *when does a spatial map for time develop in the brain*? A spatial map, intended as the spatial magnitude map, is available from birth. The structure of the representation of time would develop from a spatial-analogic form for action and simple comparison operations to a spatial-conceptual ordered and linear form for complex cognitive (syntactic, semantic and context-dependent) operations on time. The spatial meaning of temporal words, the maturation of numerical cognition and the reading/writing habits would play a crucial role in the development of compositional rules needed for ordinality and linearity of conceptual time representation. Following our review, linearity and ordinality of number cognition necessary for time representation would be available after 5 years-old age and the spatial meanings of time concepts would be functional between 8 years-old and 10 years-old age (as graphically represented in Figure 2). Further studies are necessary to support the presented proposals particularly on the cultural factors that favor the development of linearity and ordinality of the MTL for metaphor concepts of time. Formulating models on the trajectory and the functioning of mental representations development should help to study and project procedural interventions on neuro-cognitive deficits in these systems. The literature demonstrating tight relationships among spatial abilities, learning disabilities, numerical cognition and time cognition at all levels is abundant (Newcombe et al., 2015, for a recent review). Future studies oriented on disambiguating on the presented proposals and doubts should be useful for learning and educational researches.

## Author Contributions

BM and AM equally contributed to the development and conceptualization of the work, to the revision process and to the final approval for submission.

## Conflict of Interest Statement

The authors declare that the research was conducted in the absence of any commercial or financial relationships that could be construed as a potential conflict of interest.
